# Formononetin Improves the Survival of Random Skin Flaps Through PI3K/Akt-Mediated Nrf2 Antioxidant Defense System

**DOI:** 10.3389/fphar.2022.901498

**Published:** 2022-05-19

**Authors:** Haoliang Li, Renhao Jiang, Lejing Lou, Chao Jia, Linfang Zou, Mochuan Chen

**Affiliations:** ^1^ Department of Orthopaedics, The Second Affiliated Hospital, Yuying Children’s Hospital of Wenzhou Medical University, Wenzhou, China; ^2^ Zhejiang Provincial Key Laboratory of Orthopaedics, Wenzhou, China; ^3^ The Second Clinical Medical College of Wenzhou Medical University, Wenzhou, China; ^4^ Department of Respiratory and Critical Care Medicine, The First Affiliated Hospital of Wenzhou Medical University, Wenzhou, China

**Keywords:** random-pattern flap, formononetin, angiogenesis, inflammation, oxidative stress, PI3K/AKT/Nrf2 signaling pathway

## Abstract

Random-pattern skin flap is widely used in plastic and reconstructive surgery. However, its clinical effect is limited by ischemia necrosis occurs at the distal part of flap. Previous studies have proved that the protective effect of formononetin was associated with its antioxidant, anti-inflammatory ability. However, further research is still needed on the effect of formononetin on flap viability. The purpose of our study was to investigate the effect of formononetin on flap survival and the underlying mechanisms. Two doses (25 mg/kg, 50 mg/kg)of formononetin were administered for seven consecutive days on flap model. Flap tissues were collected on postoperative day 7. Our results revealed that formononetin promoted skin flap viability in a dose-dependent manner. Using immunohistochemical staining and western blot, we found that formononetin significantly reduced oxidative stress and inflammation. Hematoxylin and eosin (H and E) staining, laser Doppler images and immunofluorescence staining showed the enhancement of angiogenesis after formononetin treatment. Mechanistically, we demonstrated that the antioxidation of formononetin was mediated by activation and nuclear translocation of nuclear factor-E2-related factor 2 (Nrf2), while down-regulating cytoplasmic Kelch-like ECH-associated protein 1 (Keap1) expression. Co-treatment with formononetin and LY294002 (15 mg/kg), a potent Phosphatidylinositol-3-kinase (PI3K) inhibitor, which aborted nuclear Nrf2 expression and phosphorylated Akt, indicating that formononetin-mediated Nrf2 activation was related to PI3K/Akt pathway. Overall, our findings revealed that formononetin increased angiogenesis, reduced oxidative stress and inflammation, thus promoting flap survival. We highlighted the antioxidant effects of formononetin since the Nrf2 system was activated. Therefore, formononetin might be a promising candidate drug that can enhance survival of skin flaps.

## Introduction

Random skin flap is a method widely applicated to repair skin defects caused by refractory wounds (Sinha et al., 2012), accidental trauma damage (Volk et al., 2019), cancer excisions (Paradela et al., 2010), and diabetes mellitus (Weng et al., 2018). However, flap necrosis, a common postoperative complication, often leads to serious consequences, thus limiting its clinical application. The blood supply of the flap generally depends on the vascular ganglia within the flap’s pedicle bed and angiogenesis from the pedicle to the distal end.(Lin et al., 2018). After the random flap model is established, there is always little angiogenesis at the distal end of the flap, resulting in necrosis (Toutain et al., 2009). In addition, the restoration of blood flow after neovascularization may cause ischemia-reperfusion injury (IRI) (Reichenberger et al., 2012). Reactive oxygen species (ROS) are key influencing factors in the pathogenesis of IRI, which may cause internal environment chaos accompanied by oxidative stress (Rao et al., 2021). Hypoxia can induce an inflammatory response that can cause secondary damage to severely ischemic flap tissue (Plock et al., 2007). Another research found that flap inflammation occurred by activating nuclear factor kappa B (NF-κB) signaling pathway to release inflammatory factors such as interleukin-6 (IL-6) and tumor necrosis factor-α (TNF-α), which mediate the inflammatory response after IR and induces apoptosis (Liu et al., 2019). Thus, treatment of random skin flap necrosis may set about promoting angiogenesis, attenuating oxidative stress and inflammation.

Formononetin (FMNT) is one of the natural isoflavones isolated from Chinese medical herb Astragalus membranaceus (Fisch.) which is a typical plant estrogen with an estrogen-like effect and a small side effect (Wu et al., 2020). Previous research has indicated that FMNT had multiple pharmacological properties such as anti-oxidant (Aladaileh et al., 2019), anti-inflammatory (Kim et al., 2019), anti-hypoxia/ischemia injury (Wang et al., 2020), protecting cardiovascular system (Liang et al., 2019). However, the effect of FMNT on skin flap is still unclear. Recent study shown that FMNT prevented oxidized low-density lipoprotein (ox-LDL)-induced inflammatory response, oxidative stress and apoptosis in human vascular endothelial cells (HUVECs). Upregulation of miR-223 abrogates NOD-like receptor pyrin domain containing 3(NLRP3) inflammasome-mediated pyroptosis to attenuate ox-LDL-induced cell death in HUVECs (Zhang et al., 2021). Furthermore, previous research indicated that FMNT induced endothelial cell migration and promoted angiogenesis via an estrogen receptor alpha-promoted Rho-associated protein kinase (ROCK) pathway (Li et al., 2015). Therefore, we hypothesized that FMNT may enhance random skin flap survival.

Nrf2, a Cap-n-Collar family member protein of the basic leucine zipper, plays a major role in the cellular antioxidant process (Bellezza et al., 2018). Researchers indicated that Nrf2 was a prominent transcription factor which could regulate antioxidant phase II detoxification enzymes (Natarajan et al., 2010). Kelch-like ECH-associated protein 1 (Keap1) regulated intracellular Nrf2 expression. In addition, Nrf2 activated its downstream target genes such as superoxide dismutase (SOD), glutamate cysteine ligase catalytic/modifier (GCLc/GCLm), heme oxygenase-1 (HO-1), thioredoxin reductase (TrxR) and NAD(P)H quinone oxidoreductase-1 (NQO1) (Zhang et al., 2013). However, there are few studies on the regulatory mechanism of FMNT. According to published research, we assumed that FMNT might downregulate oxidative stress and inflammation through PI3K/AKT mediated Nrf2 antioxidant defense system. The purpose of this study was to demonstrate its effect on flap survival and to explore its associated mechanisms.

## Materials and Methods

### Animals

A total of sixty ten-week-old, wild-type male mice (C57BL/6, 20–30 g) were sourced from Shanghai Animal Center of the Chinese Academy of Sciences. Animal treatment and care was carried out in accordance with the Laboratory Animal Use and Care Guidelines of the National Institutes of Health. All procedures were approved by the Animal Care and Use Committee of Wenzhou Medical University (wydw2021-0213). All animals were treated to the best of their ability to minimize animal pain. The mice were randomly divided into four groups: the control group (*n* = 15), the low-dose FMNT group (*n* = 15), the high-dose FMNT group (*n* = 15) and the FMNT + LY294002 group (*n* = 15).

### Reagents and Antibodies

Formononetin (C16H12O4, purity ≥99%) and LY294002 were procured from Sigma-Aldrich Chemical Company (Milwaukee, WI, United States). Primary antibodies against NQO1, TrxR1, HO-1, Keap1 IL-1β, TNF-α and Lamin B were from Abcam (Cambridge, UK); antibodies against GCLc and GCLm were purchased from Boster Biological Technology (Wuhan, China); antibodies against Nrf2, GAPDH, PI3K, P-Pi3k, AKT, and P-AKT were purchased from Cell Signaling Technologies (Danvers, MA, United States); antibodies directed vascular endothelial growth factor (VEGF) and SOD2 were obtained from Affinity Biosciences (Cincinnati, OH, United States). Diaminobenzidine (DAB) developer, pentobarbital sodium, and the H and E Kit were procured from Solarbio Science and Technology (Beijing, China).

### Flap Animal Model

Firstly, we anesthetized all of the animal with 1% (w/v) pentobarbital sodium via intraperitoneal injection (50 mg/kg). After anesthesia, a caudal-based random-pattern flap (size 1.5 × 4.5 cm^2^) was raised from the back of the mouse, which beneath the panniculus carnosus (Lin et al., 2017). Next, the left and right sacral arteries were completely removed. Finally, the separated flap was immediately inserted into the donor bed and sutured with 4-0 nonabsorbable silk suture as previously described (Wu et al., 2019). The flap was evenly divided into three sections from the distal part to the pedicle: area I (proximal), area II (intermediate), area III (distal zones). Because the distal portion of flap in control group was almost completely necrotic, we could’t get useful tissue. The proximal part of flap was normal in all groups. The most significant difference is in the middle part of the flap. Therefore, we only obtained the tissue to research from area II.

### Drug Administration

All animal were divided evenly into four groups. The low-dose FMNT group (FMNT-L) received FMNT 25 mg/kg, the high-dose FMNT group (FMNT-H) and the high-dose FMNT＋LY294002 group (FMNT-H + LY294002) received FMNT 50 mg/kg once daily, while the control group received the same amount of physiological saline once daily (Yi et al., 2020). Mice were given intragastric administration of FMNT for 7 days. In the FMNT-H + LY294002 group, Akt inhibitor LY294002 was injected intraperitoneally at a dose of 15 mg/kg each 30 min before FMNT administration (dose). After 7 days, all animals were sedated by sodium pentobarbital (overdose) and then the flap tissue was harvested from area II of the flaps.

### Evaluation of Flaps Survival

On postoperative day 7, random flap survival after surgery was estimated from images of random flaps. Changes in the flap, such as hair condition appearance, texture, color, were observed on postoperative day 7 (POD 7). The surviving area was evaluated via Image-Pro Plus imaging (version 6.0; Media Cybernetics, Silver spring, MD, United States). We used this calculation to measure the percentage of viable area:

Percentage viable(%)=extent of viable area/whole flap.

### Determination of SOD and GPx Levels

On POD 7, five mice were randomly selected from each group to obtain tissue samples. A 0.5 cm × 0.5 cm flap tissue sample was isolated from the center of area II; The muscle layer of the flap was removed and the remaining tissue was treated with an ice-water bath. The homogenate was then centrifuged and the supernatant was retained. We used xanthine oxidase to measure SOD and dithiobis nitrobenzoic acid method to measure glutathione peroxidase (GSH-Px) activities.

### Laser Doppler Blood Flow Imaging

The blood supply of the flaps was assessed by Laser Doppler Blood Flow Imaging **(**LDBF) measurements. On POD 7, we selected five mice from each group to sedated and kept them prone at room temperature. We used a laser Doppler instrument (Moor Instruments, Axminster, UK) to scan the flap for further assessment of microvascular blood flow. Laser Doppler blood flow typically provided deeper penetration, enhancing visualization of small blood vessels which below the skin surface. Blood flow was quantified and analyzed by LDI version 6.1 software (Moor Instruments). We assessed blood flow intensity by perfusion unit (PU). The rats were scanned three times equally.

### Molecular Modeling

First, we drew the molecular structure of FMNT using ChemBioDraw and minimized its energy using ChemBio3D. According to the needs of current molecular docking experiments, we downloaded the corresponding PI3K (PDB ID:3APF), Nrf2-Keap1 (PDB ID:1 × 2R) and Akt (PDB ID:2F7Z) from the PDB website (https://www.rcsb.org/). After being treated via PyMoL (version 1.7.6), the lowest energy conformations for docking were determined by default parameters. And the molecular structures of FMNT, PI3K, Nrf2-Keap1 and Akt required for docking analysis have been prepared, and then the molecular docking began. The docking analysis was performed by the AutoDockTools (version 1.5.6). The eventual pictures of 3D views were performed via the PyMoL.

### Western Blotting Analysis

We harvested five tissue samples in each group from area II of the mouse skin flap and stored at -80 °C prior to sacrifice. The harvested flap tissue was first extracted with lysis buffer, and then we used BCA to determine the protein concentration. Next, we added an equal amount of 60 μg protein to a 12% (w/v) gel for electrophoresis and transferred the protein from the gel to a PVDF membranes. After blocking with 5% skimmed milk for 120°min, We incubated membrane with subsequent primary antibody overnight at 4 °C: Kepa1 (1:1000), Nrf2 (1:1000), HO1 (1:1,000), NQO1 (1:1,000), GcLc (1:1000), Lamin B (1:1000), GcLm (1:1000), TrxR1 (1:1,000), AKT (1:1,000), P-AKT (1:1,000), PI3K(1:1000), P-PI3K(1:1000), and GAPDH (1:1000). Next, the membranes were subsequently incubated with a secondary antibody for 2 h at room temperature. Finally, the protein bands were visualized using the ECL Plus Reagent Kit. Image Lab 3.0 (Bio-Rad, United States) was used to analyze band intensities.

### H and E Staining

7 days after surgery, after euthanizing the mice in each group, five samples (1 × 1 cm) from zone II were taken. Following this, we post-fixed the obtained tissue samples in 4% (v/v) paraformaldehyde for 24 h. Next, we embedded the samples in paraffin for sectioning. Sections were cut to 4 mm thickness and placed on poly-l-lysine-coated glass slides. Then we used them for H and E Staining. We evaluated microvascular density (MVD) of skin flaps via observing the HE stained tissue under a light microscope with ×200 magnification. This was for assessing the condition of endogenous angiogenesis.

### Immunohistochemistry

Firstly, we deparaffinized and rehydrated five sections from region II in each group. Next, we blocked the sections in 10% (v/v) bovine serum albumin in PBS for 1 h, then we incubated them against IL-1β (1:100), TNF-α (1:200), HO-1 (1:100), and SOD2 (1:100) overnight at 4°C overnight. After that, we incubated with secondary antibody which conjugated with HRP as previously described (Wu et al., 2019). What’s more, we used a DAB detection kit to stain those sections and counterstain with hematoxylin. The images were taken under a light microscope (Olympus). Integral absorbance of IL-1β, TNF-α, HO-1, and SOD2-positive blood vessels was calculated using Image-Pro Plus software (Media Cybernetics).

### Immunofluorescence

Five sections in each group were selected to be deparaffinized in xylene and then rehydrated in graded ethanol. After washing, sections were first soaked in sodium citrate buffer (10.2 mM) for 20 min and then treated with 0.1% (v/v) PBS-Triton X-100 for 30 min. After that, we incubated the sections with a primary antibody against VEGF (1:200) at 4°C overnight. Next, the sections were washed and incubated with FITC-conjugated secondary antibody as previously described (Wu et al., 2019). We used a fluorescence microscope (Olympus) to visualize the sections.

### Statistical Analyses

We performed statistical analysis on the data using SPSS version 19 software (SPSS Inc, Chicago, IL, United States). All data have been indicated as mean ± SEM. We used independent samples two-tailed, unpaired *t*-test to compare means between the two groups. *p* < 0.05 was considered significant.

## Results

### FMNT Promoted Survival of Skin Flap

**FIGURE 1:**
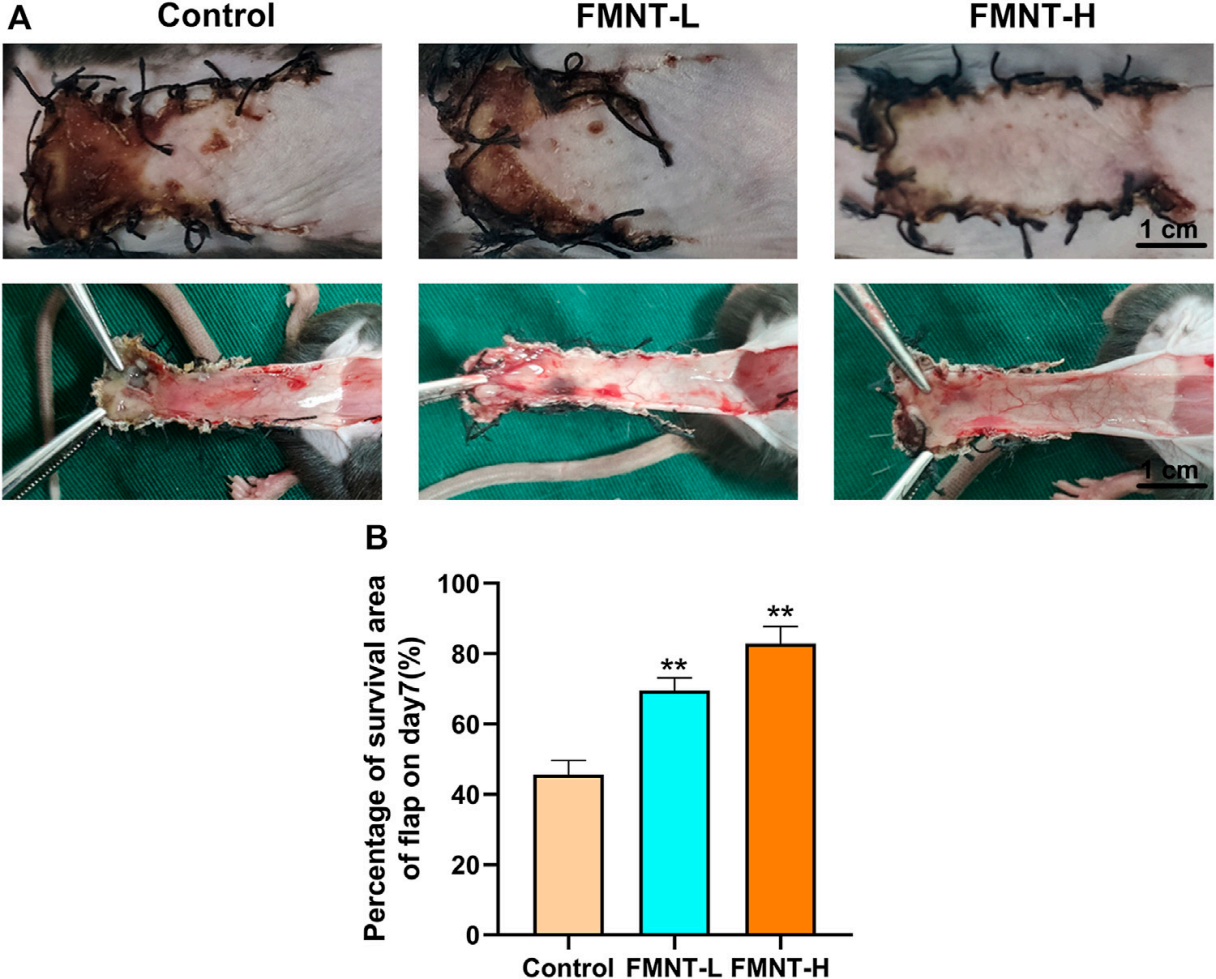
**(A)** Macroscopic view of a random skin flap on the back of a rat on day 7 after surgery (external and after flap lift) (scale bar: 1 cm) **(B)** Survival rates of the three groups of flaps. All data represent mean ± SD. **p ≤ 0.01 compared to the control group.

Necrotic flap tissue progressed after completing the operation with a clear demarcation between necrotic and viable areas on day 7. The necrotic tissue was black, hard texture with an uneven surface and had no hair growth or bleeding in the incision tissue (Figure 1A). Flaps in surviving areas of skin were pale red, soft in texture, and densely hairy. Flap examination indicated that there were staggered capillary network in surviving area (Figure 1A).

Severe necrosis was revealed via macroscopic observation in the control group. The total area of flap necrosis in the control group was significantly larger than that in the FMNT groups. The control showed significantly reduced flap survival rate (45.67 ± 2.10%) than the FMNT-L group (69.51 ± 2.30%) and FMNT-H group (82.83 ± 2.80%) (Figure 1B).

### FMNT had Anti-oxidative Stress Function in Skin Flaps

**FIGURE 2:**
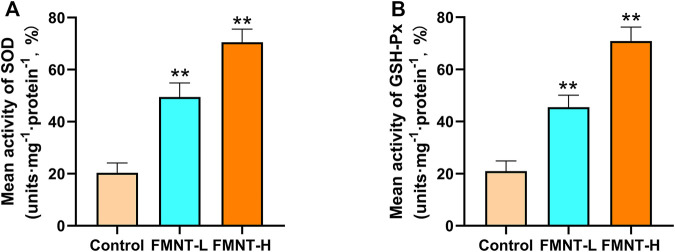
FMNT ameliorated oxidative stress in the random pattern skin flaps **(A)** The activities of SOD among three groups **(B)** Average GSH-Px activities in rat skin flap tissue. All data represent mean ± SD. **p ≤ 0.01 compared to the control group.

Oxidative stress plays an important role in skin flap necrosis. In order to find out whether FMNT had the ability to resist oxidative stress, indicators like SOD and GSH-Px were carried out to identify the level of oxidative stress. The average SOD activities of the control, FMNT-L, and FMNT-H groups were 20.33 ± 2.59, 49.53 ± 6.70, and 70.52 ± 7.00 U mg protein−1 (Figure 2A), while the GSH-Px of the control, FMNT-L, and FMNT-H groups were 21.35 ± 3.59, 45.41 ± 6.13, and 70.87 ± 7.26 U mg protein−1 (Figure 2B), respectively, proving that FMNT treatment substantially enhanced the SOD and GSH-Px activity.

### FMNT Reduced Inflammatory Cytokine Expression

**FIGURE 3:**
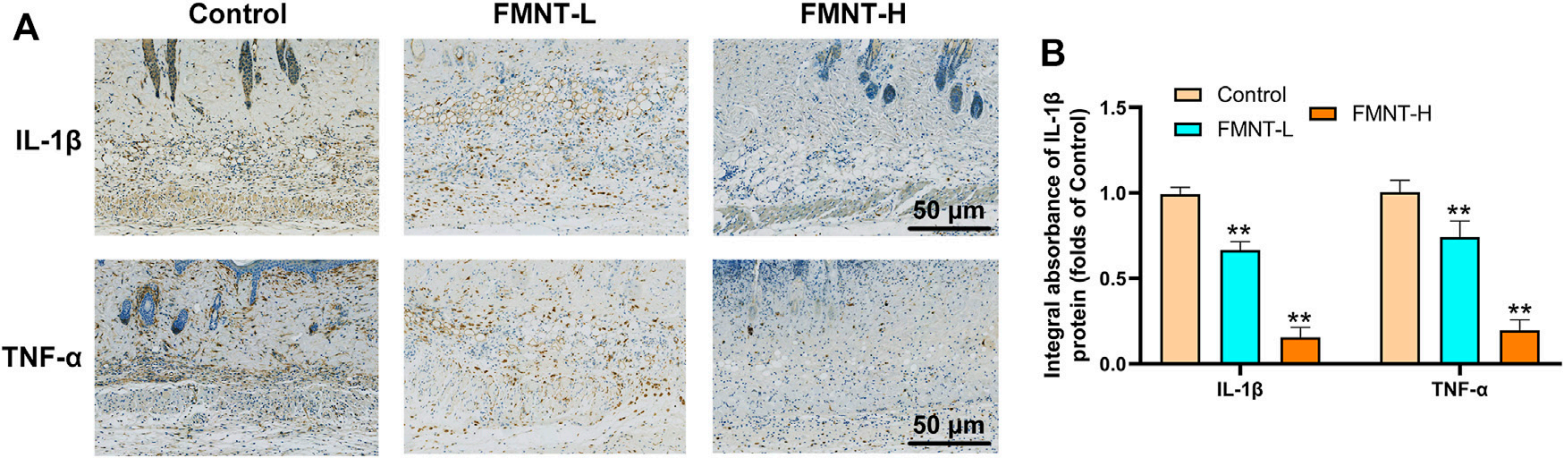
**(A)** The expressions of IL-1β and TNF-α were detected by immunohistochemistry in the tissue of the area II of the three groups of skin flaps (scan bar: 50 μm) **(B)** IHC of IL-1β and TNF-α mainly expressed in vascular endothelial and stromal cells in flaps. All data represent mean ± SD. **p ≤ 0.01 compared to the control group.

To determine the anti-inflammatory effects of FMNT on the necrosis of skin flaps, the immunohistochemical staining was used to evaluate the expression of proinflammatory cytokines (Figure 3A). The levels of IL-1β expression were reduced in the FMNT-H and FMNT-L groups than the control group (Figure 3B). In addition, the FMNT-H and FMNT-L groups showed significantly lower levels of TNF-α expression than the control group (Figure 3B). Taken together, FMNT pretreatment could abolish these inflammatory responses in a dose-dependent manner.

### FMNT Improved Flap Microcirculation and Increases Angiogenesis

**FIGURE 4:**
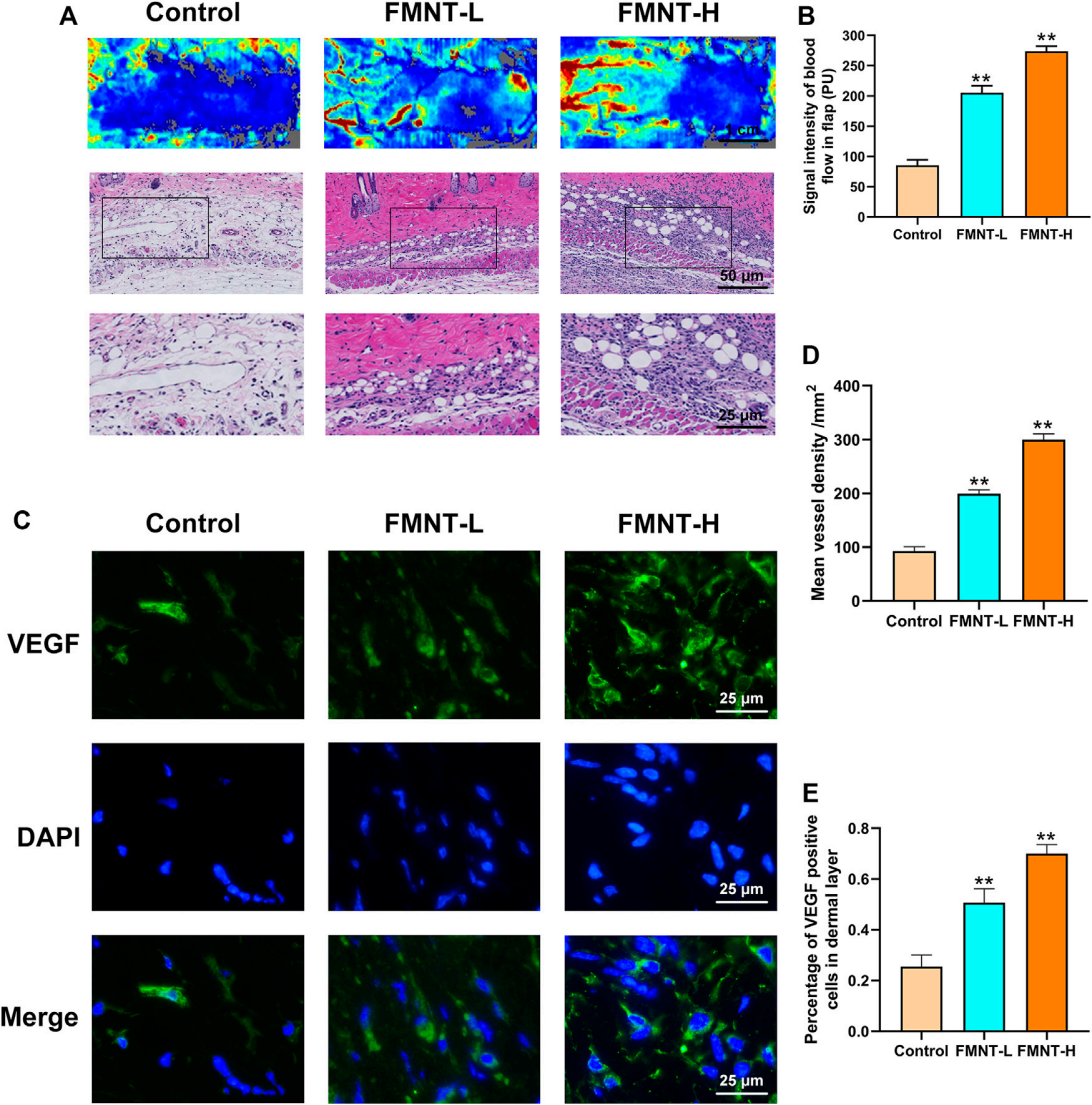
FMNT increased blood vessels in flaps **(A)** Laser Doppler images show blood perfusion in the flap (scale bar: 1 cm) The H&E staining between three groups showing the vessels (scale bar: 50 and 25 μm) **(B)** Signal intensity of blood flow in skin flaps among the three groups **(C)** The representative fluorescence image of VEGF with DAPI (nuclei) (scale bar: 25 μm) **(D)** Mean vessel density of mice skin flap tissue **(E)** The percentage of VEGF positive cells in dermal layer of skin flaps. All data represent mean ± SD. **p ≤ 0.01 compared to the control group.

Firstly, our Laser Doppler images indicated that the FMNT-H and FMNT-L groups had more neovascularization and vascular anastomosis than the control group, suggesting that FMNT improved blood microcirculation in the distal flap (Figure 4A). In addition, we used an optical microscope to observe the H and E-stained paraffin sections (Figure 4A). The signal intensity of blood flow in FMNT-H group was 299.83 ± 10.58/mm^2^, which was significantly higher than both the FMNT-L group (199.35 ± 9.07/mm2) and control group (92.57 ± 6.93/mm2) (Figure 4B). The mean vessel density was remarkably higher in the FMNT-H and FMNT-L groups in comparison to the control group (Figure 4D). To further investigate whether FMNT upregulates angiogenesis of skin flaps, we used IHC to measure the expression of VEGF (a protein associated with angiogenesis) (Figure 4C). In Immunofluorescence, VEGF expression in the FMNT-H and FMNT-L groups was higher than the control group (Figure 4E). The results indicated that FMNT improved flap microcirculation and increased angiogenesis.

### Effects of FMNT on Nrf2 Nuclear Localization

**FIGURE 5:**
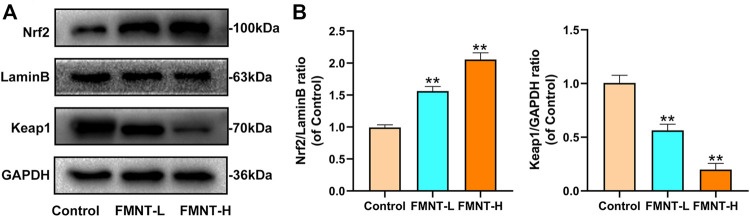
**(A)** The protein expression of Nrf2, LaminB and Keap1 was detected by western blot **(B)** The Nrf2/Lamin B1 ratio and the Keap1/GADPH ratio was determined. All data represent mean ± SD. **p ≤ 0.01 compared to the control group.

Keap1 inhibits the release of Nrf2, which activates antioxidant/phase II detoxification enzymes when Nrf2 is released into the nucleus. To confirm that the nuclear accumulation of Nrf2 by FMNT is an effective potent Nrf2 activator, the level on Nrf2 and Keap1 were detected. As indicated in Figures 5A,B, FMNT, in an incremental dose, increased nuclear Nrf2 expression and reduced cytoplasmic Keap1 expression (Figures 5A, B). Therefore, these findings suggested that FMNT effectively inhibited the oxidative processes associated with Nrf2 activation after application to the skin flap.

### Effects of FMNT on Antioxidant/phase-II Detoxification Enzymes

**FIGURE 6:**
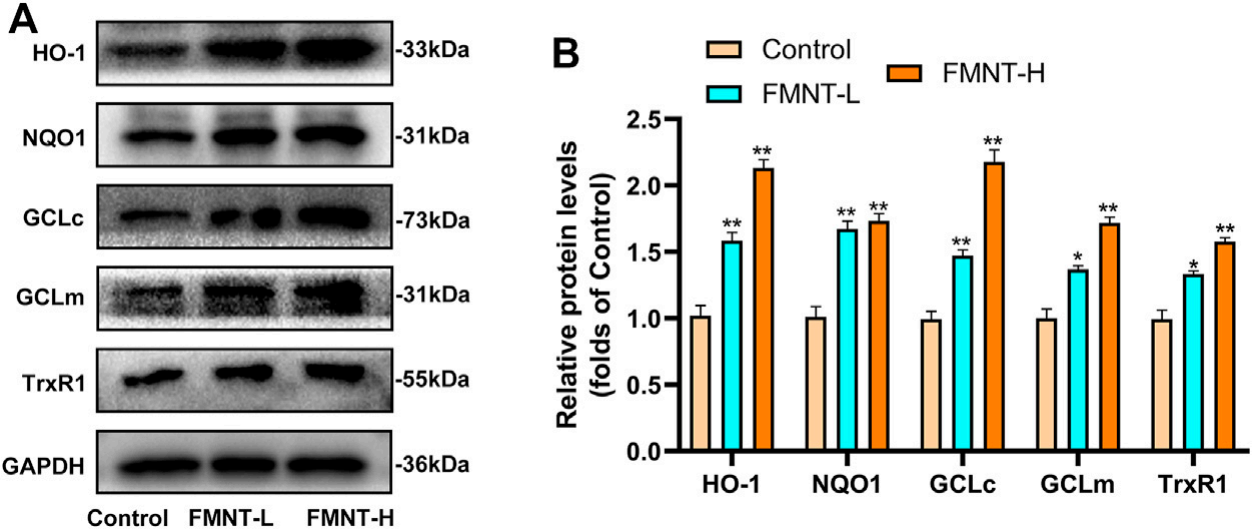
**(A)** The protein expression of HO-1, NQO1, GCLc, GCLm, TrxR1 and Keap1 was detected by western blot **(B)** The expressing levels of proteins relate to antioxidant/phase II detoxification enzymes. All data represent mean ± SD. *p ≤ 0.05 and **p ≤ 0.01 compared to the control group.

Nrf2 exerts its transcriptional function through the expression of its downstream target antioxidant/phase II detoxification enzymes (Serafini et al., 2019). The effect of FMNT on antioxidant/phase II detoxification enzymes were also observed via western blotting (Figure 6A). The expression of antioxidant/phase II detoxification enzymes, including HO-1, NQO1, GCLc, GCLm, and TrxR, were significantly increased after pretreatment with FMNT (Figure 6B). The results demonstrated that FMNT could exert potent antioxidant effects through the Nrf2-driven antioxidant defense system.

### Effects of FMNT on PI3K/Akt/Nrf2 Signaling Pathway and Downstream Antioxidant Enzymes

**FIGURE 7:**
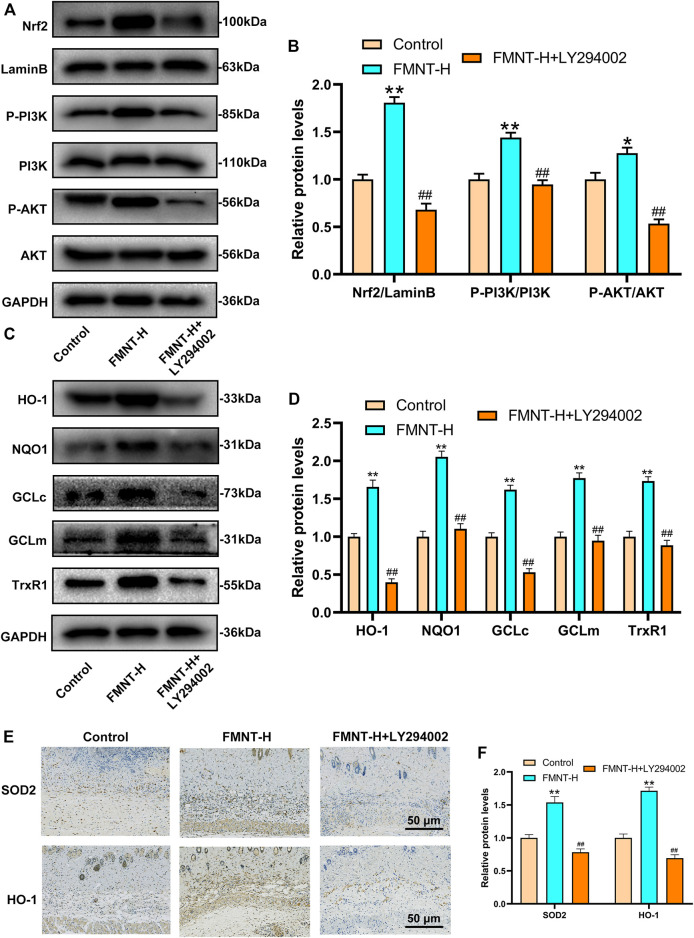
**(A,B)** Western blot along with its quantification revealed that the level of P-PI3K, P-Akt and Nrf2 as treated above **(C,D)** Western blot along with its quantification revealed that the level of HO-1, NQO1, GCLc, GCLm and TrxR1 **(E,F)** The IHC result of SOD2 and HO-1 expression in skin flaps (scale bar: 50 μm) **p ≤ 0.01 compared to the control group; ##*p* ≤ 0.01 compared to the FMNT-H group.

Research had found that the PI3K/Akt pathway was related to the regulation of Nrf2(Zhuang et al., 2019). In order to find out the mechanism by which FMNT enhances Nrf2 nuclear localization, we evaluated the activation of the PI3K/Akt pathway. LY294002 is a potent PI3K inhibitor that inhibits the expression of downstream targets such as Nrf2 and Akt. As is indicated in Figures 7A, B, the expression of nuclear Nrf2, phosphorylated PI3K and phosphorylated Akt in the FMNT-H group was elevated than control group and the FMNT-H + LY294002 group. FMNT significantly upregulated Nrf2 compared to the control group. (Figures 7A, B). To explore the antioxidant effect of FMNT through the PI3K/Akt/Nrf2 pathway, we examined the expression of downstream antioxidant enzymes of PI3K/Akt/Nrf2. The results indicated that the expression of the antioxidant enzymes such as HO-1, NQO1, GCLc, GCLm, and TrxR were remarkably decreased upon FMNT + LY294002 group than that with FMNT alone (Figures 7C, D). Moreover, the IHC result indicated that FMNT promoted the expression of SOD2 and HO-1, while decreased through LY294002 (Figures 7E, F).

### Molecular Docking Interaction of FMNT With PI3K, Akt, and Nrf2-Keap1

**FIGURE 8:**
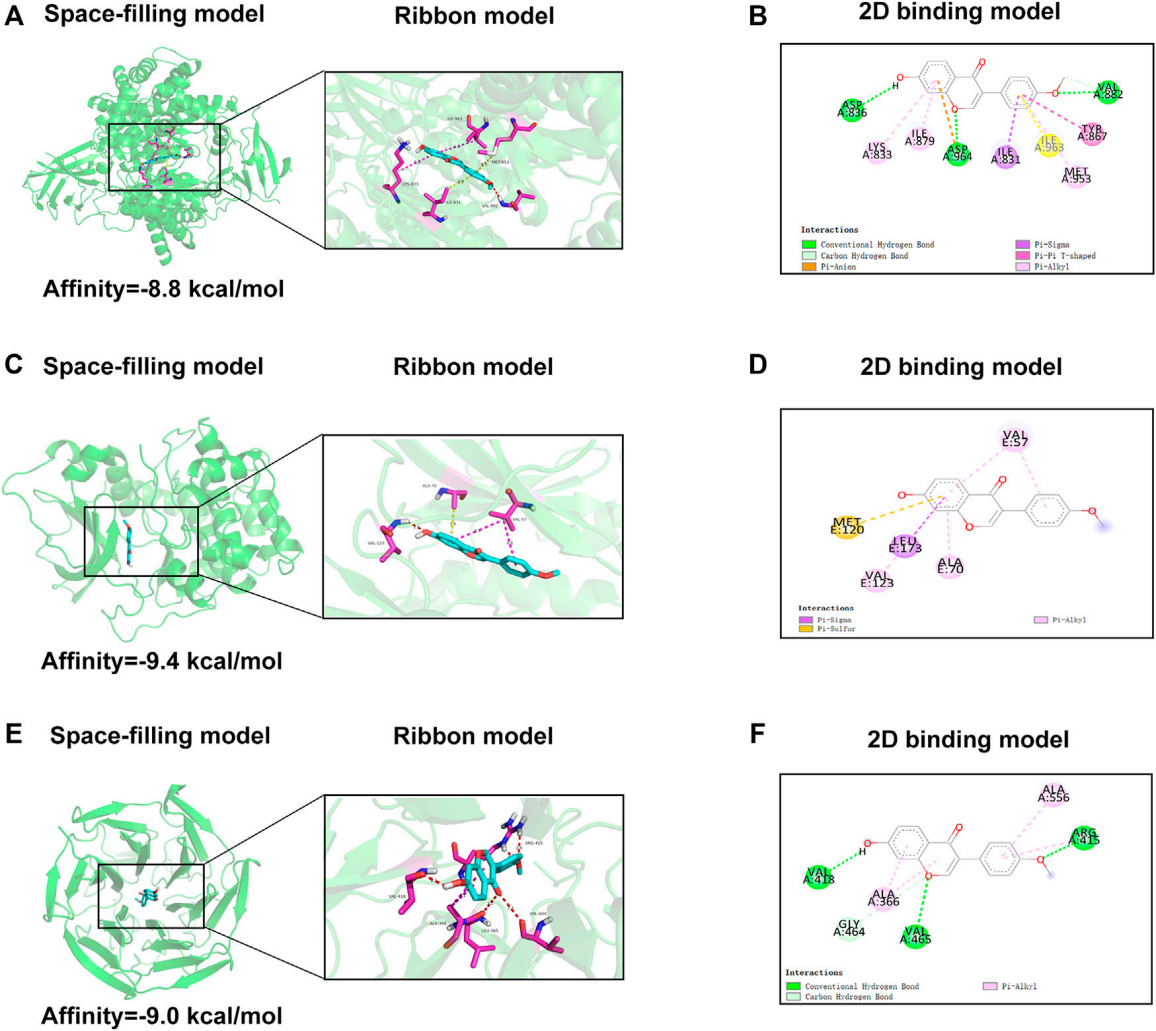
**(A,C,E)** The space-filling models and the ribbon models of FMNT with PI3K, Akt and Nrf2-Keap1 complex **(B,D,F)** The 2D binding models between FMNT and PI3K, Akt and Nrf2-Keap1 complex.

To further explore the underlying mechanism of FMNT, we applied molecular docking analysis to examine proteins that may be regulated by FMNT. We first examined related proteins of the PI3K pathway. The results of the assessment of the ability of FMNT to bind to PI3K were shown in Figure 8A, while the lowest binding energy between FMNT and PI3K was -8.8 kcal/mol. The results indicated that the interaction between FMNT and PI3K-related proteins via conventional hydrogen bonds, carbon hydrogen bonds, Pi-anion, Pi-sigma, pi-pi T-shaped and pi-Alkyl, which contained nine potential active sites (ASP836, LYS833, ILE879, ASP964, ILE831, ILE963, MET953, TYR867, VAL882) (Figure 8B).

Then, molecular docking of FMNT with the Akt-related proteins was performed. The docking results showed the lowest binding energy between FMNT and AKT was -9.4 kcal/mol (Figure 8C). Furthermore, FMNT formed pi-sigma with LEU173, pi-alkyl with VAL57, VAL123, ALA70 and pi-sulfur with MET120 (Figure 8D).

The docking simulation results in Figure 8E indicated that the lowest binding energy between FMNT and Nrf2-Keap1 was -9.0 kcal/mol. What’s more, FMNT formed conventional hydrogen bonds with VAL418, VAL465, ARG415, pi-alkyl with ALA366, ALA556 and carbon hydrogen bond with GLY464 (Figure 8F).

## Discussion

Random-pattern skin flap is one of the most common and effective surgical techniques for repairing skin defects (Cai et al., 2020). However, distal flap necrosis as a postoperative complication severely limits the clinical application of flap surgery (Pirozzi et al., 2013). Since adequately effective drugs to prevent distal flap necrosis have not yet emerged, there is an urgent need for more therapeutic drugs to prevent flap necrosis (Chen et al., 2018). As a drug with anti-inflammatory and antioxidant functions, FMNT has a great therapeutic effect on a variety of diseases (Machado Dutra et al., 2021). Our present study provided novel evidence indicating that FMNT increased flap survival through activating Nrf2-mediated antioxidant system. Therefore, we highlighted FMNT’s potential for clinical use to improve outcomes of ischemic flaps and reveal FMNT as a novel Nrf2 activator for further research.

FMNT is an isoflavone from plant estrogen groups that exhibit a wide range of physiological roles of health through estrogen dependent and independent mechanisms (Danciu et al., 2018). There have been a number of studies revealed the effect of FMNT in different diseases. FMNT reduced diabetic neuropathy via reducing oxidative stress and increasing expression of sirtuin one and nerve growth factor (NGF) (Oza and Kulkarni, 2020). In addition, researchers found that FMNT induces vasorelaxation by regulating the PI3K/PTEN/Akt signaling pathway (Li T. et al., 2018). However, there are few studies focused on FMNT in flap models. Our results showed that FMNT enhanced the survival of random pattern skin flaps through the PI3K/Akt-mediated Nrf2 antioxidant defense system.

Angiogenesis involves disruption of pre-existing blood vessels, mitosis, endothelial cell migration, and maturation of new capillaries (Mukwaya et al., 2019). In our study, H&E and laser Doppler blood flow for skin flap tissues indicated that the microvessel number in the dermis of ischemic flaps was significantly higher in high-dose FMNT group compared to the low-dose FMNT group and the control group. VEGF is a key factor involves in inducing mitosis of endothelial cells (Xin et al., 2016). In our study, the result of immunofluorescence showed that FMNT promoted VEGF expression. These results indicated that FMNT enhanced angiogenesis and the microcirculation of skin flaps.

IRI induced oxidative stress in skin flaps which result in tissue damage (An et al., 2020). ROS reacted with the plasma membrane in the early stage of oxidative stress and then triggered the destruction of cells (Lesiow et al., 2019). The previous study showed that the enzymes related to antioxidant activity played an significant role in inhibiting oxidative stress (Lei et al., 2016). SOD catalyzed the dismutation of superoxide into oxygen and H2O2, which is important in antioxidant function. Moreover, GSH-Px downregulated free H2O2 to water and lipid hydroperoxide to the corresponding alcohol (Li et al., 2013). Our study demonstrated that the expression of SOD and GSH-Px were enhanced in the FMNT groups. Furthermore, the expression of inflammatory cytokines such as IL-1β and TNF-α were greatly reduced in the FMNT groups compared to the control group. These results indicated that FMNT inhibited oxidative stress and inflammation effectively.

In order to further clarified the mechanism of action of FMNT and how to promote flap survival, we also explored the upstream mechanism of antioxidant activity. Nrf2 is a redox regulator that plays a central role in the inducible expression of antioxidant/phase II detoxification enzymes (Zhang et al., 2015). Upon oxidative stress, the Nrf2 is inhibited by Keap1-mediated proteasomal degradation under basal conditions, resulting in low Nrf2 protein levels (Baird and Yamamoto, 2020). Our study revealed that FMNT enhanced the nuclear localization of Nrf2, which effectively enhanced antioxidant process.

Antioxidant/phase II detoxification enzymes are involved in flap protection by regulating intracellular oxidative processes (Rasti Ardakani et al., 2017). GCLC and GCLM govern the production of the endogenous antioxidant GSH(Weldy et al., 2012). HO-1 catalyzes the degradation of heme and releases antioxidants such as free iron, carbon monoxide and biliverdin (Loboda et al., 2016). Many studies have reported that NQO1 protected cells against oxidative stress by reducing the toxic 1,4-benzoquinone to the less reactive hydroquinone via two-electron reduction (Siew et al., 2012). The main function of TrxR enzyme is to sustain the stability of thioredoxin system via catalyzing the NADPH-dependent reduction of thioredoxin (Zhang et al., 2017). Therefore, we believed that the upregulation of HO-1, GCLs, TrxR and NQO1 expression might lead to a potent antioxidant function in skin flaps. Moreover, previous study had shown that Nrf2 involved in regulating the expression of antioxidant genes that exerted anti-inflammatory functions (Saha et al., 2020), suggesting the anti-inflammatory effects of FMNT might be partly attributed to Nrf2-mediated activation of HO-1.

Researchers found that PI3K/Akt pathway is an upstream regulator of Nrf2(Sun et al., 2019). Previous studies had found a close relationship between the PI3K/Akt pathway and the Nrf2 antioxidant pathway. Firstly. Research has indicated that activation of PI3K/Akt pathway triggered nuclear translocation of Nrf2(Hu et al., 2018), then enhanced phase II enzyme (such as HO-1 and NQO1) expression and thus reflected the antioxidant effect of AST on photoreceptor cells (Lai et al., 2020). Besides, PI3K/Akt pathway can cooperate with Nrf2 antioxidant system to inhibit Aβ-triggered oxidative damage which is the molecular mechanism for the neuroprotective effect of Sargahydroquinoic acid (Yoon et al., 2021). Our study shown that PI3K/Akt activation was positively correlated with nuclear Nrf2. Furthermore, the PI3K inhibitor LY294002 significantly reversed the Nrf2 expression and its downstream genes which was influenced via FMNT. The result demonstrated that FMNT increased the expression of antioxidant/phase II detoxification enzymes via promoting the nuclear Nrf2 and that the entire process is mediated via promoting the PI3K/Akt signal pathway. Studies have shown that there are two mechanisms for the activation of Nrf2. First, it changes the conformation of keap1 by oxidizing cysteine residues in Keap1, while inducing disconnection of the Nrf2-Keap1 complex (Li et al., 2020). The other mechanism involves multiple upstream kinases such as PI3K/Akt pathway, which promoted Nrf2 activation by inducing Nrf2 phosphorylation and nuclear translocation (Li S. T. et al., 2018).

**FIGURE 9:**
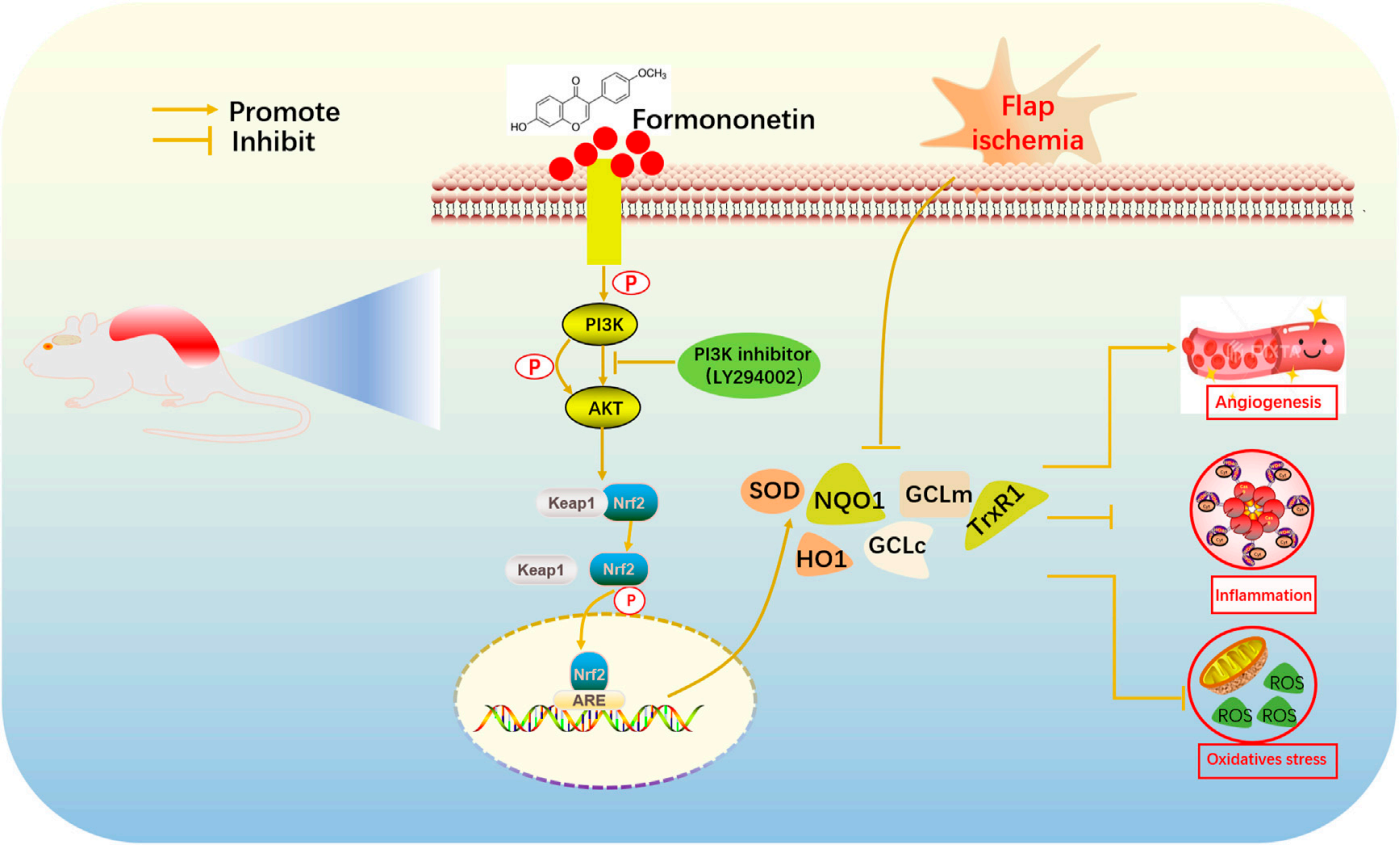
Molecular mechanisms of antioxidant property by FMNT.

Western blot analysis revealed that FMNT enhanced Nrf2 and its downstream antioxidant enzymes through mechanisms including the PI3K/Akt signal pathway and the Nrf2-Keap1 complex disruption. The molecular docking result revealed that hydroxyl and carboxyl groups were decisive for FMNT biological function (Figure 8). Binding of FMNT to Keap1 prevented the production of the Nrf2-Keap1 complex and stabilized Nrf2, resulting in releasing Nrf2, thereby stimulating the transcription of antioxidant genes. In addition, FMNT exerted its biological properties by directly binding to PI3K through hydrogen and hydrophobic bonds. Our findings demonstrated that FMNT could prevent oxidative stress in skin flaps via promoting the Nrf2-driven antioxidant defense system, and active Nrf2 was regulated by PI3K/Akt signal pathway, as indicated in Figure 9.

However, there are still some problems that need deeper research. First, the study only evaluates short-term effects of FMNT, while its long-term effect is still unknown. Furthermore, this experiment cannot prove that FMNT is also effective on human skin flaps, so we still need further study in large animal models like pig or rabbit prove the effect of FMNT for clinical use. What’s more, optimal drug dose, timing, median effective dose (ED50) and duration of management are not clear. These should be investigated in the future. Nevertheless, current research shows that FMNT involves in improving the survival of random skin flaps which is a safe, economical, effective and natural compound with potential clinical application prospects.

## Conclusion

In summary, this study proves that FMNT promoted the viability of random skin flaps by promoting angiogenesis, reducing oxidative stress and inflammation. Moreover, we found that FMNT suppressed oxidative damage through PI3K/Akt-mediated Nrf2 antioxidant defense system. Furthermore, molecule docking result showed that FMNT had strong affinity for PI3K, Akt and Nrf2-keap1. Our study reveals the potential therapeutic benefits of FMNT in the random skin flaps.

## Data Availability

The original contributions presented in the study are included in the article/Supplementary Material, further inquiries can be directed to the corresponding author.
